# Ischemic Stroke and SARS-CoV-2 Infection: The Bidirectional Pathology and Risk Morbidities

**DOI:** 10.3390/neurolint14020032

**Published:** 2022-04-24

**Authors:** Vishal Chavda, Bipin Chaurasia, Alessandro Fiorindi, Giuseppe E. Umana, Bingwei Lu, Nicola Montemurro

**Affiliations:** 1 Department of Pathology, Stanford School of Medicine, Stanford University Medical Center, San Francisco, CA 94305, USA; chavdavishal2@gmail.com (V.C.); bingwei@stanford.edu (B.L.); 2 Department of Neurosurgery, Bhawani Hospital and Research Center, Birgunj 44300, Nepal; trozexa@gmail.com; 3 Neurosurgery, SpedaliCivili, Department of Medical and Surgical Specialties, Radiological Sciences and Public Health, University of Brescia, 25100 Brescia, Italy; alessandro.fiorindi@gmail.com; 4 Department of Neurosurgery, Trauma and Gamma-Knife Center, Cannizzaro Hospital, 95100 Catania, Italy; umana.nch@gmail.com; 5 Department of Neurosurgery, Azienda Ospedaliera Universitaria Pisana (AOUP), University of Pisa, 56100 Pisa, Italy

**Keywords:** COVID-19, anti-coagulants, ischemia, SARS-CoV-2, therapeutics, neurology, neurosurgery, stroke

## Abstract

Stroke is a fatal morbidity that needs emergency medical admission and immediate medical attention. COVID-19 ischemic brain damage is closely associated with common neurological symptoms, which are extremely difficult to treat medically, and risk factors. We performed literature research about COVID-19 and ischemia in *PubMed, MEDLINE*, and *Scopus* for this current narrative review. We discovered parallel manifestations of SARS-CoV-19 infection and brain ischemia risk factors. In published papers, we discovered a similar but complex pathophysiology of SARS-CoV-2 infection and stroke pathology. A patient with other systemic co-morbidities, such as diabetes, hypertension, or any respiratory disease, has a fatal combination in intensive care management when infected with SARS-CoV-19. Furthermore, due to their shared risk factors, COVID-19 and stroke are a lethal combination for medical management to treat. In this review, we discuss shared pathophysiology, adjuvant risk factors, challenges, and advancements in stroke-associated COVID-19 therapeutics.

## 1. Introduction

The current COVID-19 (coronavirus disease 2019) pandemic, also known as the severe acute respiratory syndrome (SARS-CoV-2), was caused by a novel coronavirus (severe acute respiratory syndrome coronavirus 2; SARS-CoV-2). Wuhan, China, was the site of the first reports of an atypical pneumonia outbreak with an unknown etiology in December 2019 [1]. Initially, local clinicians diagnosed it as virus-based pneumonia or virus-induced pneumonia and respiratory illness based on the symptoms and clinical diagnosis. Cases continued to emerge quickly, and the etiological agent, a novel coronavirus strain (2019-nCoV), was identified by 2020 [2]. A new coronavirus was discovered in the bronchoalveolar lavage fluid/saliva of infected patients at Wuhan Jinyintan Hospital. After a virological research investigation, it was discovered to be an enveloped, positive-sense, single-stranded RNA virus, a member of the Coronavirus family, the same virus family associated with severe acute respiratory syndrome (SARS) and Middle East respiratory syndrome (MERS) [3]. Later, WHO designated a virus with many new names: Wuhan coronavirus, 2019-nCoV, SARS-CoV-2, and novel coronavirus (nCoV)—based on the epidemic’s original location: Wuhan City, Hubei Province, China in 2019. The disease is classified as COVID-19 disease, and symptoms range from asymptomatic to severe. Epidemiologic clinical studies and case reports from around the world have shown that SARS-CoV-2 can cause significant respiratory, hepatic, and neurological complications [4,5,6,7,8]. Aging, diabetes, hypertension, neutrophilia, lymphocytopenia, high inflammatory indicators, and coagulopathy are also major causes of death and breathing distress in COVID-19 patients. Since the beginning of the global pandemic, the most common COVID-19 symptoms have been fever, cough, dyspnea, sore throat, nausea, vomiting, anorexia, and fatigue [9]. Dyspnea, a high breathing rate, and low blood oxygen saturation levels are all symptoms of severe COVID-19. Some coronavirus patients, particularly those over 65 and those with chronic medical conditions such as diabetes, hypertension, cancer, and pulmonary asthma, may be at risk for acute respiratory distress syndrome and organ dysfunction [10,11]. Critical disease symptoms include respiratory failure, septic shock, and multiple organ failure (MOF) [12]. Accumulating evidence also indicated that one-third of the affected population experienced neurological symptoms, such as dizziness, headache, myalgia, impaired consciousness, ageusia, anosmia, encephalitis, and cerebrovascular stroke, with nerve pain, visual impairment, seizure, occipital neuralgia, and ataxia being uncommon [1,13,14,15,16,17]. SARS-CoV-2 has the potential to affect both the central nervous system (CNS) and the peripheral nervous system (PNS), resulting in neurological symptoms and complications. SARS-CoV-2 can enter the CNS directly, via retrograde neuronal transport within vagal nerve afferents, or via transcribrial route or through infiltrate with infected leukocytes [18,19]. Furthermore, SARS-CoV-2 can compromise the blood–brain barrier (BBB) integrity by targeting angiotensin-converting enzyme 2 (ACE-2) receptors found on the endothelial cells of blood vessels in the brain, promoting BBB permeability and viral entry into the CNS [20,21]. COVID-19 may cause ischemic stroke through hypercoagulability, severe inflammation, renin-angiotensin-aldosterone system dysfunction, cardiac dysfunction, and the effects of severe respiratory illness, according to this. In addition to traditional stroke mechanisms, hypercoagulability, severe inflammation, renin-angiotensin-aldosterone system (RAAS) dysfunction, cardiac dysfunction, and the consequences of severe respiratory illness are potential mechanisms of ischemic stroke associated with COVID-19. Patients who have excessive coagulation, blood stasis, and endothelial damage are also predisposed to thrombosis and, as a result, stroke. Stroke is currently the world’s second leading cause of death, according to the latest WHO report, and one of the common health issues in the developed countries. It is a serious neurological complication that has been observed during the ongoing COVID-19 pandemic caused by SARS-CoV-2. Blockage of an artery as well as rupturing or injury to the artery itself can result in stroke, which can be classified as ischemic, hemorrhagic, or traumatic stroke. Stroke incidence has been observed in approximately 1 to 3% of COVID-19 inpatients, indicating high odds of stroke in more severe COVID-19 subjects.

## 2. Materials and Methods

A *PubMed*, *MEDLINE*, and *Scopus* review was carried out to identify all studies dealing with ischemic stroke in COVID-19 patients. The following search terms were used from database inception to December 2020: stroke, ischemic stroke, SARS-CoV-2 infection, COVID-19, SARS-CoV-2. A total of 3182 articles, including those listed in the references of the retrieved studies, were found originally. We then excluded the following items: all publications not dealing with ischemic stroke and COVID-19 or neuropathology; all studies different from original articles (e.g., case report/case series, letters, commentaries, etc.); all preclinical studies or research performed on animals or cell cultures; non-English written papers; and any other publication that did not comply with the goal of the present review. Further relevant references were identified from the bibliography of extracted articles as needed. After this process, a total of 88 studies were included in this review.

## 3. Results

### 3.1. Epidemiology

Stroke appears to be uncommon in the COVID-19 setting [22,23]. In terms of prevalence, ischemic stroke outnumbers hemorrhagic stroke. The rates of ischemic stroke and intracranial hemorrhage associated with COVID-19 in hospitalized patients ranged from 0.4 to 2.7 percent and 0.2 to 0.9 percent, respectively. Furthermore, cryptogenic stroke accounts for 10–30% of all ischemic strokes and is directly related to COVID-19 disease [24]. The rates of COVID-19-related cerebrovascular incidents are primarily based on retrospective cohort studies of COVID-19-treated patients hospitalized in various epicenters around the world, including China, Spain, and the United States [24,25,26,27,28,29,30,31,32]. These reports cover a wide range of populations in terms of disease severity, co-morbidities, and follow-up periods, all of which can lead to stroke. Nonetheless, the risk of stroke varies depending on the severity of COVID-19. According to early case reports, the risk is 1% for patients with minor illnesses, but it may be as high as 6% for patients in intensive care [14]. Stroke usually occurs 1–3 weeks after ongoing COVID-19 symptoms, but in a small number of reported patients, stroke was the first symptom that led to hospitalization [33,34]. Two patients in a group of ten COVID-positive patients treated with mechanical thrombectomy for major artery blockage exhibited no signs of COVID-19 prior to the onset of the stroke [34]. In separate research of 32 hospitalized patients with ischemic stroke and COVID-19, it was observed that stroke was the primary reason for admission in 44% of the cases [24]. COVID-19, on the other hand, appears to be an independent risk factor for in-hospital stroke [33].

### 3.2. Risk Factors

In COVID-19 patients, the risk of ischemic stroke is increasing. Ischemic stroke is the most common cerebrovascular complication reported in COVID-19 patients. Furthermore, there have been reports of cerebral sinus thrombosis, intracerebral hemorrhage, and subarachnoid hemorrhage [26,35,36]. The mechanisms of stroke in COVID-19 are proposed to be multifaceted, involving both specific pathophysiological features of the SARS-CoV-2 virus, such as endothelial activation and thrombosis, as well as nonspecific effects of impaired coagulation and inflammation, which are superimposed on preexisting risk factors [24,37]. In addition, several cases of cryptogenic stroke in COVID-19 patients suggest that SARS-CoV-2 can cause stroke via atypical or novel mechanisms, such as COVID-19 infection-associated hypercoagulability and a pro-inflammatory state [38,39,40]. Analysis of the clot waveform has shown hypercoagulability that precedes or coincides with severe disease [38,40]. Hypercoagulability, together with a systemic inflammatory response to viral infection, could lead to the formation of macro- and micro-thrombi, ultimately causing cerebrovascular accidents [38]. A thromboelastography study of the coagulation profile in critical COVID-19 patients yielded consistency with a hypercoagulative state. A substantial increase in factor VIII has been hypothesized to be associated with COVID-19-related hypercoagulability [39]. Activation of the complement pathway, inflammatory cytokines, as well as cytoplasmic microparticles originating from platelets or lymphocytes could also induce hypercoagulative state [38,39].

Age, gender, ethnicity, and genetics are traditional stroke risk factors, as are vascular risk factors such as hypertension, dyslipidemia, diabetes, cardiovascular disease, cardioembolism, atrial fibrillation, obesity, inflammation, and infection [41,42]. Extracorporeal membrane oxygenation (ECMO) is required in conditions such as hypercoagulability, thrombosis, and coagulopathy. When comparing COVID-19-related stroke cases to older patients with vascular risk factors, data analysis from several reports shows that younger patients with low prevalence of standard stroke risk factors and elevated markers of inflammation (ferritin) and coagulation outnumber COVID-19-related stroke cases [16,23,32,36,43,44,45,46,47]. As a result, endotheliitis caused by direct viral invasion and inflammation caused by cytokine storm may be the COVID-19-peculiar mechanisms underlying such anomalies [48,49]. Serious infections, such as influenza, sepsis, and minor respiratory and urinary tract infections, have been linked to an increase in inflammation and subsequent thrombosis, which can lead to an acute stroke. However, preliminary data show that, when compared to influenza, COVID-19 is linked to a 7.6 percent increased risk of ischemic stroke [50]. Furthermore, because extremely elevated D-dimer levels (a marker of clot turnover) have been confirmed in a significant number of patients within the first few weeks of disease, especially in more severely affected individuals; a hypercoagulable state is highly anticipated in COVID-19 patients [3,7]. However, recent studies have found D-dimer levels are only slightly increased in COVID-19 patients, especially when compared to septic patients [51]. Although the presence of D-dimer suggests fibrinolytic pathways are intact and actively dissolving (lysing) fibrin, the discovery of fibrin deposits in lungs and other organs suggests dysregulation of the balance in fibrin forming (i.e., thrombin generation) and fibrin-dissolving (i.e., plasmin generation) pathways is a major aspect of COVID-19 pathogenesis [51]. Certain ischemic stroke patients had significantly higher D-dimer levels, and aggressive thrombosis was observed [52]. Furthermore, compared to non-COVID stroke patients, COVID-19 patients may have a higher rate of early re-occlusion after mechanical thrombectomy due to underlying hypercoagulability [45]. Thrombotic risk is likely to appear in COVID patients, according to preliminary reports, because anticardiolipin, antiphospholipid, and beta-2 glycoprotein-1 antibodies have been linked to COVID-related stroke [53]. Very recently, it was shown that NETs can be found in brain tissue from ischemic stroke patients and that NETs contribute to ischemic stroke brain damage, showing the pathological role of NETs in the acute setting of ischemic stroke and their contribution to long-term results [54]. Several papers reported that neutrophils are primed to make NETs in the setting of COVID and contribute to immunothrombosis in COVID-19 acute respiratory distress syndrome [55,56,57,58]. Results support the hypothesis that NETs may represent drivers of severe pulmonary complications of COVID-19, may explain the prothrombotic clinical presentations in COVID-19, and suggest that NET-targeting approaches could be considered for the treatment of uncontrolled tissue-damaging and thrombotic responses in COVID-19 [55,58]. Furthermore, antiphospholipid antibodies seem to play a key role in this process. Therefore, an interesting hypothesis is that COVID-19 exacerbates the neutrophil-like response contributing to worse stroke outcomes [59]. Patients with antiphospholipid syndrome form durable autoantibodies to phospholipids and phospholipid-binding proteins, such as prothrombin and β2 glycoprotein I (β2GPI). These autoantibodies engage cell surfaces, where they activate endothelial cells, platelets, and neutrophils, thereby tipping the blood endothelium interface toward thrombosis [59]. Higher titers of phospholipid-binding proteins were associated with neutrophil hyperactivity, including the release of neutrophil extracellular traps (NETs), higher platelet counts, more severe respiratory disease, and lower clinical estimated glomerular filtration rate [59]. SARS-CoV-2 infection has also been linked to arrhythmia, heart failure, and myocardial infarction, all of which can lead to a cardioembolic stroke [60]. In line with this, COVID-19-related coagulopathy has been linked to spontaneous intraparenchymal and cortical subarachnoid hemorrhage, suggesting that anticoagulants may be a promising treatment option for such patients [26,37]. Furthermore, COVID-19 ECMO patients have an increased risk of ischemic stroke and infrequent intracranial hemorrhage due to air embolism [61]. This points to a complicated relationship between traditional risk factors, COVID-19 infection, and stroke and raises concerns about relatively younger patients who suffer a stroke during the ongoing pandemic. In contrast, the underlying cause-and-effect relationship remains unknown [62,63].

### 3.3. COVID-19 Infection and Neuro-Pathogenesis

**Figure 1:**
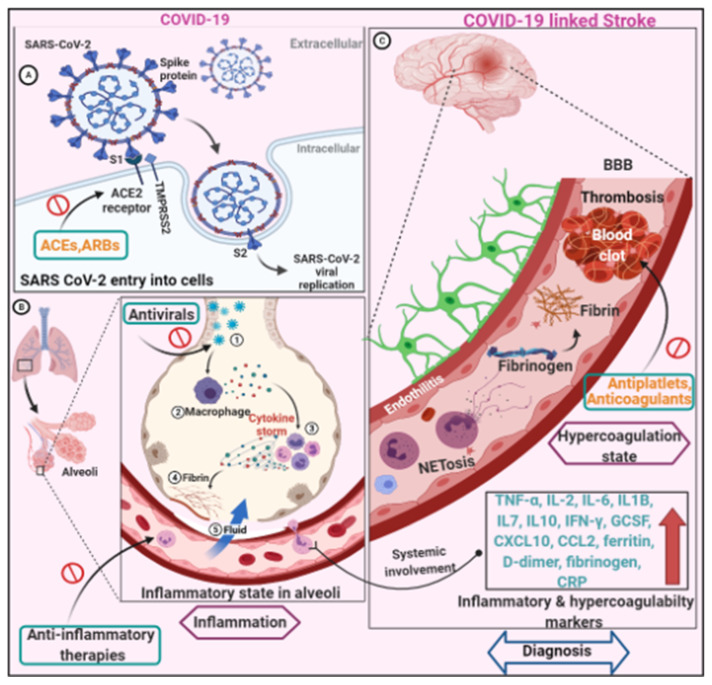
Schematic illustration of pathomechanisms related to COVID-19 infection and COVID-19-linked stroke. (**A**) The mechanism by which the SARS-CoV-2 enters cells is depicted. Angiotensin-converting enzyme 2 (ACE2), the main protein, interacts with the spike protein of SARS-CoV-2, allowing the virus to enter the cell. A difference in ACE2 levels may make people more susceptible to SARS-CoV-2 infection. (**B**) An inflammatory state in the pulmonary alveoli, which leads to pulmonary tissue edema and, eventually, systemic involvement of pro-inflammatory cytokines. (**C**) Thrombotic complication (stroke) caused by COVID-19 infection. Microvascular and macrovascular thrombosis complications result from anticipated intravascular thrombosis, including cerebral insult and stroke [67]. As a result of potent local and systemic cytokine production, platelets become activated and interact with neutrophils, enhancing the process of neutrophil extracellular trap (NET)osis. As a result, it may increase thrombin production and fibrin deposition. Excess fibrin deposition and fibrinolysis shutdown result in intravascular thrombosis and hypercoagulability, which eventually lead to clinical thromboembolic stroke complication. As a result, inflammatory and hypercoagulability markers are elevated in infected individuals, assisting in prognosis and diagnosis. Thrombolytics, anticoagulants, anti-inflammatory therapy, antivirals, angiotensin-converting enzyme (ACE) inhibitors, and angiotensin receptor blockers are among the potential therapies used to treat COVID-19 and COVID-19-associated stroke (ARBs).

COVID-19-related neurologic complications are caused by a variety of mechanisms that are multifactorial. Neurologic symptoms can develop because of both the virus’s direct effects and the body’s systemic response to infection [15]. Strokes in patients with COVID-19 may be due to usual causes such as atherosclerosis, hypertension, and atrial fibrillation. It seems likely that these COVID-19-related mechanisms would also increase the risk of stroke in infected persons who harbor the more conventional stroke risk factors [49]. The three main mechanisms appear to be responsible for the occurrence of ischemic strokes in COVID-19 are hypercoagulable state, vasculitis, and cardiomyopathy. While the pathogenesis of hemorrhagic strokes in the setting of COVID-19 has not been fully elucidated, it is possible that the affinity of the SARSCoV-2 for ACE2 receptors, which are expressed in endothelial and arterial smooth muscle cells in the brain, allows the virus to damage intracranial arteries, causing vessel wall rupture [64]. Direct viral invasion in the nervous system, neurologic injury from systemic dysfunction, RAAS dysfunction, and immune dysfunction are the distinct pathomechanics that lead to neuropathogenesis in COVID-stroke patients. Hypoxemia, which is common in COVID-19 patients, is likely to aggravate vascular and metabolic abnormalities in the brain, eventually leading to ischemic insult. Few autopsy studies have found evidence of direct viral invasion of the nervous system, but the severity of neuropathological findings has nothing to do with these findings [65,66]. However, due to the scarcity of evidence, it is unclear whether SARS-CoV-2 directly infects the cerebral vessels. Aside from that, a few postmortem studies have revealed that SARS-CoV-2 can directly invade endothelial cells via plausible pulmonary, cardiac, renal, liver, and bowel endotheliitis and is associated with inflammation and apoptosis [67,68]. However, this remains debatable given that viral particles seen in electron microscopy of kidney vessel endothelium may have been normal structures or artefacts [69,70]. Recently, Hottz et al. [71] identified a subset of inflammatory monocytes presenting high CD16 and low HLA-DR expression as the subset mainly interacting with platelets during severe COVID-19. Although no pathological tests were performed, some case reports suggested that multifocal ischemic and hemorrhagic lesions could be associated with endothelial implication, microthrombosis, or vasculitis in small vessels [72,73]. Another important pathophysiological mechanism of COVID-19 infection is maladaptive activity of the RAAS system. The membrane-bound protein angiotensin converting enzyme 2 allows SARS-CoV-2 to enter cells (ACE2). ACE2 converts angiotensin II to angiotensin-(1–7), a vasodilator with antiproliferative, antifibrotic, and antihypertensive properties [74,75]. The SARS-CoV-2 virus may cause secondary cardiomyopathies and cerebrovascular effects by binding to ACE2. A cytokine storm-mediated systemic immune response to SARS-CoV-2 is a critical mechanism for COVID-19-related clinical manifestations [76,77]. TNF-, IL-2, IL-6, IL1B, IL7, IL10, IFN-, GCSF, CXCL10, CCL2, ferritin, D-dimer, fibrinogen, leukocytosis, and C-reactive protein (CRP) levels are significantly higher in critically ill COVID-19 patients [78,79,80]. For the majority of these cytokines and chemokines, including those that are typically associated with macrophage activation syndrome (IL-6, IL-18, IFN-γ, TNF-α, CXCL9), the increase was less pronounced in COVID-19 critical condition than in macrophage activation syndrome patients [81]. On the other hand, some markers (i.e., IL-5, IL-7, IL-17A, CXCL8, and VEGF) were increased in critical COVID-19 patients only and not in macrophage activation syndrome [81]. Proinflammatory cytokine levels in the blood can cause confusion and alter consciousness as well as “thromboinflammation”, which raises the risk of stroke and other thrombotic events [82,83,84,85]. Furthermore, complement activation in patients with severe COVID-19 can result in thrombotic microvascular injury [86,87,88]. Besides, pro-inflammatory cytokine release may activate microglia, resulting in cerebral insult [87]. While several COVID-19-related stroke mechanisms have been proposed, one important mechanism appears to be host immune response or virus-related thrombophilia, as evidenced by elevated markers of hypercoagulability and inflammation [83] (Figure 1). COVID-19-endotheliitis could explain the systemic impaired microcirculatory function in different vascular beds and the related clinical complications [89]. Relevant to this, recent autoptic studies of COVID-19 patients have shown the presence of fibrin thrombi within distended small vessels and capillaries along with extensive extracellular fibrin deposition [90].

### 3.4. Linking of Mitochondrion Dysfunction, Ischemia, and COVID-19 Infection

**Figure 2:**
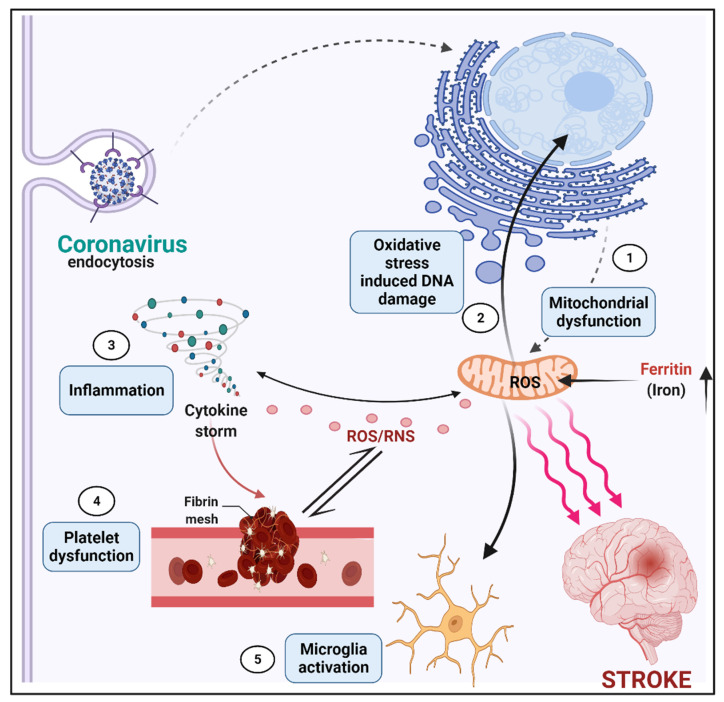
Association of COVID-19 Infection, Immunity, Mitochondria, and Ischemic Insult. It depicts the pathogenic signaling pathways in the cerebral ischemia cascade that are involved in mitochondrial function and the formation of reactive oxygen species (ROS). The downstream signaling pathways of glutamate excitotoxicity caused by ischemic stroke are depicted schematically. Excessive Ca^2+^ influx induces mitochondrial malfunction and the formation of reactive oxygen species (ROS), which leads to pathological processes as mitochondrion-dependent apoptosis, mitochondrial fission and fusion, mitophagy, DNA damage response, and inflammatory responses. A cytokine storm leads to platelet dysfunction, whose association with microglia activation, caused by mitochondrial dysfunction and ROS production, can cause ischemic stroke.

It is well-reported that COVID-19 affects the CNS in a variety of ways, and its neurological manifestations vary from individual infected [91]. COVID-19, according to new research, hijacks immune cells’ mitochondria, replicates within mitochondrial structures, and affects mitochondrial dynamics, causing cell death. COVID-19-infected cells’ mitochondria are extremely vulnerable, and this vulnerability increases with age. Mitochondria were first studied for their bioenergetic role, but they are now known to have a part in a wide range of cellular processes and signaling events. Viruses can take advantage of mitochondrial fission and fusion during host infection [92]. Fission and fusion are the vital processes of mitochondrial cell survival by producing balanced ROS production. Infected mitochondria with SARS-CoV-19 infection induce an excessive amount of ROS by increased fusion activity. which leads to too much iron storage, impaired mitophagy, and platelet apoptosis (Figure 2). This SARS-CoV-2-affected mitochondria serves as double membrane vesicle for viral entry [2]. From the initial stages of cerebral artery occlusion through the late stages of recovery, the inflammatory process plays a role in the ischemic cascade [2,5,93]. The inflammatory response encompasses both innate and adaptive immune-cell responses, possibly opening the door to novel treatment approaches [94]. The mitochondria are the center of oxidative equilibrium in the cell. A growing body of evidence links COVID-19 patients’ disease progression to a hyper-inflammatory state known as the “cytokine storm”, which involves major systemic perturbations, such as iron dysregulation manifested as hyperferritinemia linked to disease severity, as well as reactive oxygen species (ROS) production and oxidative stress [95]. The increased inflammatory/oxidative state may cause mitochondrial malfunction, which might result in platelet damage and death. Furthermore, mitochondrial oxidative stress may contribute to microbial dysbiosis by changing coagulation pathways and fueling the inflammatory/oxidative response, thus perpetuating the vicious cycle [77].

### 3.5. Diagnostic Assessment

During the pandemic, all patients with suspected stroke should have COVID-19 tested at the time of admission [96]. This recommendation is based on the fact that many stroke patients can test positive for COVID-19 despite the absence of systemic infection and clinical symptoms. When systemic COVID-19 symptoms are detected early, patients who test positively can be isolated appropriately. Given that traditional vascular risk factors and typical stroke pathophysiologic mechanisms are linked to the stroke frequency in COVID-19, the initial diagnostic approach should be the same as that used for all patients with suspected stroke. A hypercoagulability test, for example, is used to confirm stroke, and a similar approach should be used for COVID-19 patients with an unknown or defined underlying mechanism of stroke. Brain and neurovascular imaging as well as cardiac evaluation should be used to identify the underlying stroke mechanism, and treatment should be tailored to the identified mechanism. For all COVID-19 patients, regular tests include a complete blood count (CBC), platelet count, prothrombin time (PT), activated partial thromboplastin time (aPTT), fibrinogen, and D-dimer. However, the risks of arterial and venous thrombosis as well as central nervous system lesions must be evaluated in order to reduce the negative effects and death of COVID-19 victims [97].

### 3.6. Management Issues

The specific stroke treatment, along with infection control precautions, is recommended for the management of ischemic or hemorrhagic stroke in patients with suspected or confirmed COVID-19 [98,99,100]. Thrombolytics, anticoagulants, anti-inflammatory therapy, antivirals, angiotensin-converting enzyme (ACE) inhibitors, and angiotensin receptor blockers are among the potential treatments for COVID-19-related stroke patients (ARBs). Because of the risk of bleeding, the FDA-approved intravenous tissue plasminogen activator (tPA; alteplase) is primarily used in the early stages of stroke, as the patient becomes ineligible for tPA administration after 4.5 h. There are no obvious safety concerns with tPA in specific COVID-stroke patients, necessitating appropriate evaluation for thrombolytics as well as mechanical thrombectomy in such a comorbid setting [30,101]. Mechanical thrombectomy, on the other hand, has produced conflicting results in small cohort studies of COVID-19 and acute large vessel occlusion patients [16,45]. Consistent with this, COVID-19 patients may be at increased risk of re-occlusion after initial recanalization, possibly due to infectious hypercoagulability. Given the high thrombotic risk seen in COVID-19 patients, it is reasonable to begin anticoagulants as soon as possible in patients with suspected or confirmed COVID-stroke, provided the bleeding risk is tolerable. Heparin and warfarin anticoagulants, which target multiple clotting factors, may be more effective than direct oral anticoagulants (DOACs), which target a single clotting factor. More evidence will be provided by larger studies [67]. However, before deciding on antithrombotic therapy, the severity of the disease, the presence of other impending thrombotic events, and the risk of bleeding should all be considered. Anti-inflammatory therapies (Tocilizumab, IL-B antagonists) are primarily concerned with the delayed elimination of the virus, which increases the risk of secondary infections in COVID-19 patients. Anecdotal evidence suggests that corticosteroids could be used to treat COVID-19 complications; however, the role of anti-inflammatory drugs and corticosteroids in stroke is debatable. Furthermore, the first FDA-approved antiviral drug for SARS-CoV-2, Remedesvir, has no reported role in stroke [61]. COVID patients are typically prescribed ACE inhibitors or ARBs, but these medications should be stopped if they develop co-morbidities, such as hypotension or acute kidney injury, or are in the acute phase of an ischemic stroke. Although it has been suggested that COVID-19 patients who receive these agents are more likely to experience adverse side effects, observational studies have not supported this. As with any stroke patient, long-term treatment of vascular risk factors and an adequate amount of antithrombotic should be initiated for secondary stroke prevention. Finally, the increased risk of intravascular blood coagulation associated with SARS-CoV-2 can lead to stroke, which has proven to be a significant complication impeding COVID-19 management. Clinicians managing patients with suspected or confirmed SARS-CoV-2 infection during the COVID-19 pandemic should monitor these patients for potential late complications, as delayed diagnosis can lead to increased patient morbidity and mortality, as frequently reported [102,103].

### 3.7. Severity and Prognosis

According to observational studies, stroke associated with COVID-19, which causes a high rate of death and disability following ischemic stroke, is more serious than stroke without COVID-19 [104,105]. In addition, limited data show that Afro-Americans have higher mortality rates and poorer predictions of acute stroke associated with COVID-19 than other races [106]. Stroke can, however, be caused by ageing, oxidative stress, endothelial dysfunction, inflammation, or other vascular risk factors. COVID-19-induced hypoxia, hypercoagulation, and a pro-inflammatory state contribute to the occurrence, progression, and prognosis of COVID-19-related stroke [107,108]. Critically ill patients present several additional risk factors for nervous system damage. Reasons for these include deep sedation and extended muscular paralysis, bed rest for several days, and the inability to receive proper physical rehabilitation [48,49,109].

### 3.8. Preventive Measures

Acute cerebral stroke is still a potentially fatal and disabling illness, and patients who test positively for COVID-19 should seek immediate medical attention as well as optimal medical care, which has been shown to improve stroke outcomes. Universal precautions should be taken to prevent the spread of COVID-19 infection. Patients with cerebrovascular disease should keep an extra supply of medications on hand in case of a home quarantine or a disruption in supply chains. A highly safe and efficient telehealth visit is frequently performed in patients suffering from transient ischemic attack (TIA) for rapid ambulatory evaluation and in secondary prevention and rehabilitation of stroke during the COVID-19 pandemic [110,111,112,113,114,115]. However, the increased risk of stroke in the COVID-19 setting may result in a COVID-19-related widespread stroke epidemic, necessitating critical care measures to prevent a stroke epidemic in people with COVID-19 disease [97,105]. Platelets represent a potential therapeutic target for improved clinical outcomes in patients with COVID-19. Recent trials in which thromboprophylaxis was assessed for COVID-19 patients showed in noncritically ill patients with COVID-19, an initial strategy of therapeutic-dose anticoagulation with heparin increased the probability of survival to hospital discharge compared with usual-care thromboprophylaxis [116], whereas in critically ill patients with COVID-19, an initial strategy of therapeutic-dose anticoagulation with heparin did not result in a greater probability of survival to hospital discharge or a greater number of days free of cardiovascular or respiratory organ support than did usual-care pharmacologic thromboprophylaxis [117]. Berger et al. [118] evaluated the benefits and risks of adding a P2Y12 inhibitor to anticoagulant therapy among non-critically ill patients hospitalized for COVID-19, reporting no increased odds of improvement in organ-support-free days within 21 days during hospitalization. Similarly, Chow et al. [119] in their study of adults hospitalized with moderate COVID-19 showed that early aspirin use was associated with lower odds of 28-day in-hospital mortality. Further randomized clinical trials that include diverse patients with different COVID-19 clinical condition are warranted to adequately evaluate heparin and aspirin’s efficacy in patients with different risk conditions.

## 4. Conclusions

Stroke is an iniquitous SARS-CoV-2-linked neurovascular complication. Stroke has been associated with age, co-morbidities, and serious diseases in COVID-19 subjects. Although stroke is a rare complication of COVID-19, when it occurs, it frequently causes considerable morbidity and mortality. The coagulation factors and respiratory issues lead to the risk factors of stroke, i.e., hypoxic brain dead and associated neurodegeneration. The key to the minimum mortality and morbidity of patients with acute stroke is timely evaluation and hyperacute treatment. Stroke is a common neurovascular complication of SARS-CoV-2. Stroke has been linked to age, co-morbidities, and serious diseases in COVID-19 subjects. Even though stroke is a rare complication of COVID-19, it frequently results in significant morbidity and mortality. Coagulation issues and respiratory problems are risk factors for stroke, which results in hypoxic brain death and neurodegeneration. Timely evaluation and hyperacute treatment are critical in reducing mortality and morbidity in patients suffering from an acute stroke. During the current pandemic, medical teams should be aware of a wide range of COVID-19 neurological manifestations and ensure adequate isolation and protection for any suspected patients. It is critical to collect comparative data on stroke phenotypes, treatment details, and actual outcomes in patients with and without COVID-19 to better understand these emerging co-existing phenomena.
